# Deprotection Reagents in Fmoc Solid Phase Peptide Synthesis: Moving Away from Piperidine?

**DOI:** 10.3390/molecules21111542

**Published:** 2016-11-15

**Authors:** Omar F. Luna, Johana Gomez, Constanza Cárdenas, Fernando Albericio, Sergio H. Marshall, Fanny Guzmán

**Affiliations:** 1 Fraunhofer Chile Research, Santiago 7550296, Chile; omar.luna@fraunhofer.cl (O.F.L.); constanza.cardenas@pucv.cl (C.C.); sergio.marshall@pucv.cl (S.H.M.); 2 Núcleo Biotecnología Curauma, Pontificia Universidad Católica de Valparaíso, Valparaíso 2373223, Chile; johanagomezuribe@gmail.com; 3 CIBER-BBN, Networking Centre on Bioengineering, Biomaterials and Nanomedicine, University of Barcelona, Barcelona 08028, Spain; albericio@ub.edu; 4 Department of Organic Chemistry, University of Barcelona, Barcelona 08028, Spain; 5 School of Chemistry, University of KwaZulu-Natal, Durban 4001, South Africa; 6 Instituto de Biología, Pontificia Universidad Católica de Valparaíso, Valparaíso 2373223, Chile

**Keywords:** *N*-α-deprotection reagent, piperazine, piperidine, 4-methylpiperidine, solid-phase peptide synthesis

## Abstract

The deprotection step is crucial in order to secure a good quality product in Fmoc solid phase peptide synthesis. 9-Fluorenylmethoxycarbonyl (Fmoc) removal is achieved by a two-step mechanism reaction favored by the use of cyclic secondary amines; however, the efficiency of the reaction could be affected by side reactions and by-product formation. Several aspects have to be taken into consideration when selecting a deprotection reagent: its physicochemical behavior, basicity (pKa) and polarity, concentration, and time of reaction, toxicity and disposability of residues and, finally, availability of reagents. This report presents a comparison of the performance of three strategies for deprotection using microwave-assisted Fmoc peptide synthesis. Four peptide sequences were synthesized using Rink amide resin with a Liberty Blue™ automated synthesizer and 4-methylpiperidine (4MP), piperidine (PP), and piperazine (PZ) as Fmoc removal reagents. In the first instance all three reagents behaved similarly. A detailed analysis showed a correlation between the hydrophobicity and size of the peptide with the yield and purity of the obtained product. The three reagents are interchangeable, and replacement of piperidine could be advantageous regarding toxicity and reagent handling.

## 1. Introduction

The 9-fluorenylmethoxycarbonyl (Fmoc) strategy is the most used strategy in solid phase peptide synthesis (SPPS) and remains valid even forty years after its implementation [1], thanks to the constant development and improvement in reagents and strategies for the different steps [2,3,4,5].

Although it is commonly understood that coupling is the most demanding reaction in the whole synthetic process, the α-amino deprotection step is also crucial in order to secure the quality of the target peptide. Poor efficiency in deprotection will result in decreased yield and quality due to deleted residues and even capped peptides, which also generates the need of additional purification steps [1,6]. Contrary to *tert*-butoxycarbonyl (Boc) chemistry, where the α-amino deprotection is carried out in trifluoroacetic acid (TFA), which is the best solvent/reagent to disaggregate the peptide chain, Fmoc removal is carried out in *N*,*N*-dimethylformamide (DMF), which is a worse solvent to disrupt the interchain aggregation, very often favored by the presence of the own Fmoc group [6].

**Scheme 1:**
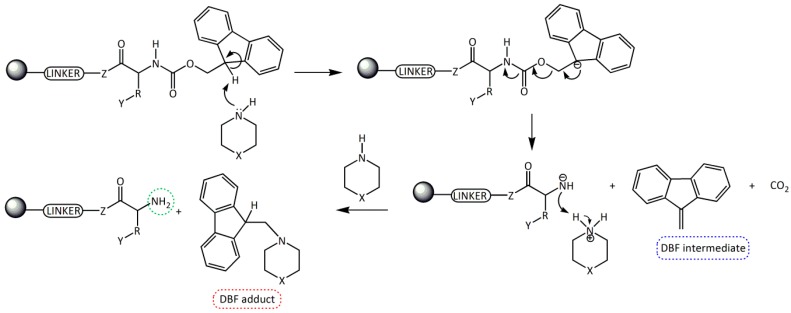
Removal of the Fmoc group by secondary amines for the formation of a free -NH_2_ in SPSS. R = amino acid side chain; Y = side chain protecting group; Z = O, NH; X = CH_2_, NH, and N(CH_3_).

Fmoc group removal in solid phase peptide synthesis (SPPS) proceeds through a two-step mechanism: the removal of the acidic proton at the 9-position of the fluorene ring system by a mild base, preferably a secondary amine, and the subsequent β-elimination that yields a highly reactive dibenzofulvene (DBF) intermediate which is immediately trapped by the secondary amine to form stable adducts (Scheme 1). These reactions work better in an electron donor and relative polar medium (DMF or *N*-methylpirrolidone [NMP]) compared to a relatively non-polar one (dichloromethane [DCM]) [6,7].

Although, the Fmoc group can be easily removed by primary amines and, less easily, by tertiary amines, the most convenient method involves the use of cyclic secondary amines due to their nucleophilicity. These amines trap very efficiently the DBF intermediate generating an adduct and, therefore, driving the deprotection step to completion [8,9,10,11]. The most used secondary amine is piperidine (pKa = 11.1, 25 °C), although one of the main reported problems with its use is the formation of aspartimide [12,13], which can be minimized by the use of other bases.

Additionally, availability is its major drawback; in fact, piperidine has a current legal status as a controlled substance regulated by the Drug Enforcement Agency (International Narcotics Control Board for 2014), because it may be used as a precursor of illegal psychotropic drugs. This fact implies that special permission is required for purchasing piperidine which, in certain institutions or countries, can cause administrative problems. In this regard, other cyclic secondary amines, 4-methyl piperidine, or piperazine (pKa = 10.78 and 9.73, 25 °C respectively) have been also proposed [8,9,10].

This report presents the comparison of three strategies for Fmoc removal using microwave-assisted peptide synthesis to investigate if the use of 4-methylpiperidine (4MP) or piperazine (PZ) is comparable in terms of efficiency with the use of piperidine (PP) and, therefore, can replace it. To this respect, this study has been carried out using four sequences in high-demand production in our laboratory, of medium-large size peptides (up to 26 residues), which allows a realistic context for making the comparison of the deprotection conditions (Table 1), using a Rink amide resin, and a Liberty Blue™ microwave automated synthesizer. Peptides were characterized by high-performance liquid chromatography (HPLC), mass spectrometry and circular dichroism in order to determine purity, presence of byproducts, and the secondary structure of the resulting peptides. In addition, deprotection kinetic assays were conducted using arginine and leucine as initial amino acids to complement and corroborate our results.

## 2. Results

### 2.1. Yield and Purity of Synthesized Peptides

Total crude yield, purity, and peptide-specific yield (as defined in Materials and Methods) were obtained according to the HPLC data analysis of the crude and purified product (Supplementary Materials Tables S1–S4 and Figures S1–S4). These results are summarized in Table 2.

Each peptide showed similar results, regardless of the deprotection reagent used. Table 2 summarizes these results.

Each peptide showed nearly the same values in total crude yield, purity, and peptide-specific yield with the three deprotection reagents. Peptide NBC1951 showed the greater variation among the piperidine and the other two deprotection reagents. PP showed the best results in purity and peptide-specific yield (the two are related), except in the case of peptide NBC112 where better yields (crude yield and peptide-specific yield) were obtained with 4MP, but better purity was obtained with PZ.

### 2.2. HPLC and Mass Spectrometry

**Figure 1:**
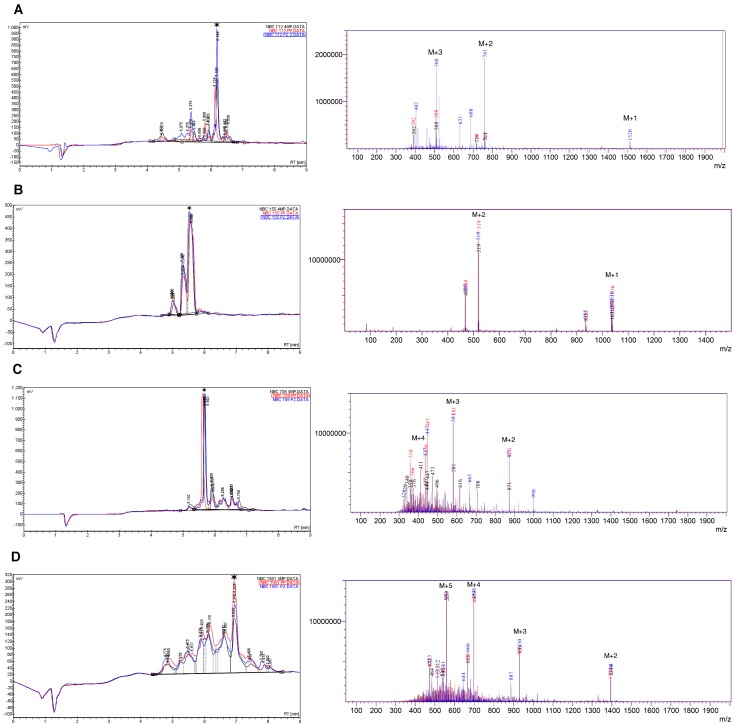
HPLC and mass spectra (ESI-MS) for crude peptides, (**A**) NBC112; (**B**) NBC155; (**C**) NBC759; and (**D**) NBC1951. The data are superimposed for the three deprotection reagents; Black: 4-Methyl piperidine (4MP), red: Piperidine (PP), and blue: Piperazine (PZ). A star indicates the main species in HPLC. The peaks marked as M + n, in the ESI spectra, indicate the peak for the corresponding n ion of the peptide (M + 1, M + 2, …, M + 5); for details see the Materials and Method section and Table 3.

Results of HPLC and mass spectrometry (electrospray ionization (ESI)-MS) for crude synthetic peptides are summarized in Figure 1 and Table 3.

As observed, the chromatograms and mass spectra for the three deprotection strategies are superimposable with regard to the main components; however, in each specific case there are differences in the number of species obtained in the crude product (Tables S1–S4).

The enrichment of the peptide through the C18 extraction columns with an acetonitrile:water gradient only allowed purification in the case of the peptide NBC155; with the other peptides enrichment occurred, but the product was accompanied by other substances (Figures S1–S4).

Matrix-assisted laser desorption/ionization-time of flight (MALDI-TOF) mass spectrometry analysis of the specific peaks in HPLC of enriched products showed specific sequence deletions, which can come from either incomplete incorporation of the deleted residues or incomplete removal of the Fmoc from the previous residue (Table 4):

(1) NBC112: Alanine and histidine deletion with 4MP and PZ with a %area higher than 10%.
(2) NBC759: Lysine deletion with a %area higher than 10% in all cases.
(3) NBC1951: Glutamine/lysine deletion with a %area higher than 10% in all cases, the two amino acids co-eluted in the chromatogram (Figure S4 peak 1).

### 2.3. Secondary Structure Characterization

**Figure 2:**
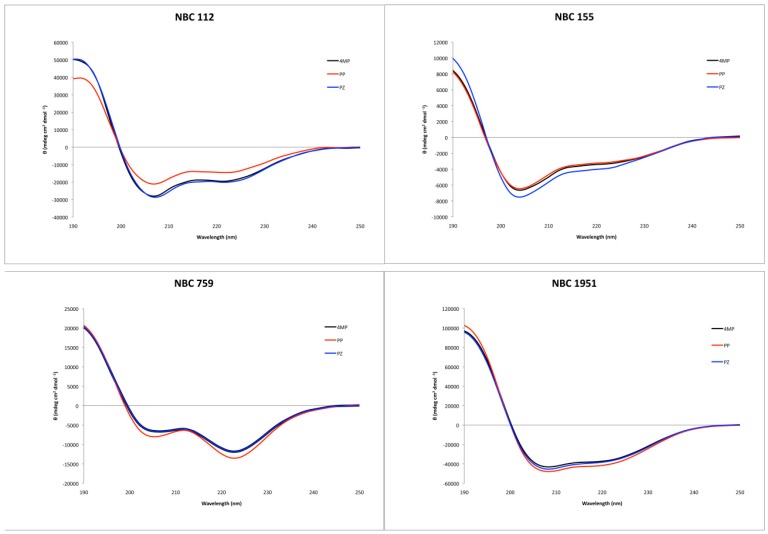
Circular dichroism spectra of the synthesized peptides, with the three deprotection reagents. Black: 4-methyl piperidine (4MP), red: piperidine (PP), and blue: piperazine (PZ).

Circular dichroism showed an alpha helix tendency for all four peptides (Figure 2), in accordance with previous reports for NBC155 and NBC1951 [16,19]. Pepfold3 server [20] prediction for the secondary structure also showed the alpha helix as the best model for all four peptides.

### 2.4. Physicochemical Properties

To complement this study some physicochemical properties related to hydrophobicity were calculated (Table S5), but only one of them, the ratio of hydrophilic residues/total residues, was meaningful in the context of the results obtained, which are presented along with a graph representation of the sequences according to the Hopp and Woods hydrophobicity scale [21] (Table 5).

### 2.5. Deprotection Kinetics

**Figure 3:**
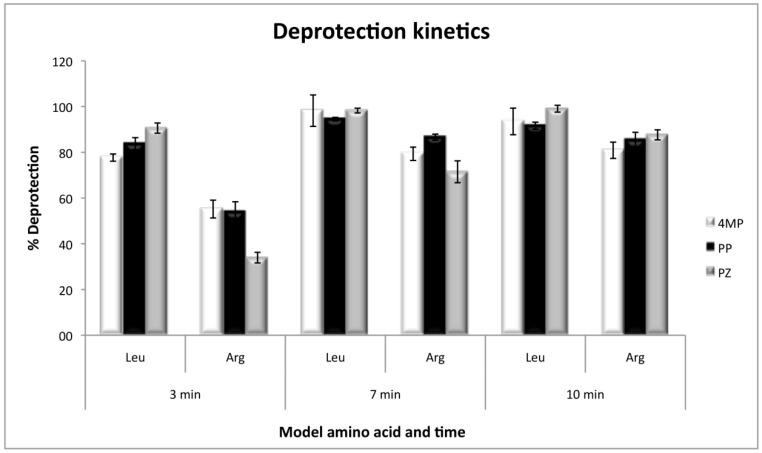
Deprotection kinetics for Fmoc-l-Leucine-OH and Fmoc-l-Arginine(Pbf)-OH as model amino acids, with the three deprotection reagents. Values were calculated as the percentage of the theoretical resin loading by measuring absorbance at 300 nm.

The kinetics of deprotection, by using Fmoc-l-Arginine(Pbf)-OH and Fmoc-l-Leucine-OH as the first coupled amino acids, showed that arginine deprotection needs a minimum of 10 minutes to be efficient, and at that time the three deprotection reagents present no significant differences. However, at short deprotection times, PZ was less efficient than 4MP and PP. On the other hand, with leucine, the deprotection was efficient with any of the three reagents, even at times as short as 3 min with 80% of efficiency and without significant differences at 7 or 10 min (Figure 3).

## 3. Discussion

According to the results obtained, any one of the reagents tested is a good choice for use in the deprotection step of Fmoc synthesis.

The yields obtained for each peptide were similar with the three reagents used, although piperidine showed a slightly higher value with three of the peptides (NBC155, NBC759, and NBC1951). It can be said that both 4MP and PZ are good options as an alternative for the removal of the Fmoc group, and may have some advantages over PP, regarding toxicity and reagent handling, according to the safety information (Table S6).

It should be noted that, in a broad context, any of the three deprotection reagents can be used, regardless of the specific peptide.

However, with a more detailed analysis, there are two related factors that should be considered: the hydrophobic-hydrophilic character of the peptide sequence and their size. The first one, according to the results obtained, plays a major role in determining the yield and purity of the product obtained. With the sequences worked in this report, it was possible to establish a direct relationship of the yield and purity of the product obtained with the hydrophilic residues/total residues ratio and size of the peptide (Table 5); based on that, peptides can be ordered as follows: NBC155 > NBC759 > NBC112 > NBC1951. This order reflects the hydrophilic character, and the size, with the last two sequences as the most hydrophobic and NBC1951 as the longest peptide.

In the case of NBC155 and NBC759 peptides, the yield and purity obtained were quite good and this correlates with the hydrophilic character of the sequence and their length; according to the experience in our laboratory these are two peptides well-behaved in SPPS.

The synthesis of the hydrophobic peptides NBC112 and NBC1951 must be optimized in the coupling steps to increase yield and purity; despite this, the deprotection results showed only minor differences among deprotection reagents.

The synthesis process was the same for all peptides, so that differences are only due to variation in the deprotection reagent used. HPLC analysis showed the presence of several species both in the crude and purified product, which could be related to deprotection, but also with the coupling step, which is highly dependent on the sequence, the specific amino acid, and its protecting group. According to the experience in our laboratory, the deletions observed in the sequences can be separated in two groups: first, those related with the coupling step, and second, those related to the deprotection step.

The sequence of the peptide NBC112 showed a hydrophobic cluster, AII, and the alanine deletion observed with PZ and 4MP is located in this cluster (Table 4 and Figure S1 peak 3). This deletion is not observed with PP, which could be due to the basicity of the reagent (PP has the highest pKa) as has been reported before for PZ in this type of sequences.

NBC759 lysine deletion (Table 4 and Figure S3 peak 2) has nearly the same area with all three reagents; the deprotection with PZ has a slightly higher %area that could be related to the lysine (K_8_) coupled just after a hydrophobic cluster in the sequence (ALA), similarly than with the NBC112. Additionally, its sequence has two pairs of consecutive lysines, and repetitive sequences are making coupling more difficult.

NBC1951 glutamine/lysine deletion (Table 4 and Figure S4 peak 1) are more likely related to incomplete coupling; the corresponding peak in the chromatograms has the same %area with the three deprotection reagents. In the case of glutamine, which carries a trityl protecting group, the steric hindrance interferes with the coupling; the lysine deletion could be related to the hydrophobic sequence before K6 (ALY) or K14 (LAW).

One important issue to be considered is related to the size of the protecting groups; in this case the deprotection of arginine, which is a difficult amino acid in peptide synthesis due to the size of its protecting group 2,2,4,6,7-pentamethyldihydrobenzofuran-5-sulfonyl (Pbf), suggests that the deprotection process should be optimized, which according to the experience in our laboratory, should be done by an extra deprotection step, without increasing the concentration of the reagent or the reaction time in order to minimize side reactions.

It can be stated that although, in general terms, the three reagents are interchangeable, the specificity could be improved according to the amino acid involved in the deprotection step, in a similar way as are described for the DX sequence involved in aspartimide formation. In our case for hydrophobic clusters (ILV) the reagent with the best performance was PP.

## 4. Materials and Methods

### 4.1. Reagents and Solvents

All Fmoc-protected amino acids, *N*,*N*-diisopropylcarbodiimide (DIC), OxymaPure^®^, and Fmoc-Rink Amide AM resin (0.6 mmol/g) were purchased from Iris Biotech GmbH (Marktredwitz, Germany). Solvents for synthesis, deprotection reagents, and cleavage reagents used were of synthesis grade and purchased from Merck KGaA (Darmstadt, Germany). Solvents and other chemicals used for high-performance liquid chromatography (HPLC), electrospray ionization (ESI) and matrix-assisted laser desorption/ionization-time of flight (MALDI-TOF) mass spectrometry (MS) and circular dichroism (CD) spectroscopy analyses were of HPLC reagent grade and purchased from Merck KGaA (Darmstadt, Germany).

### 4.2. Peptide Synthesis

Peptides sequences (Table 1) were synthesized using a Liberty Blue™ automated microwave peptide synthesizer (CEM Corp., Matthews, NC, USA) following a standard Fmoc/tBu protocol. A Rink Amide AM resin (loading 0.6 mmol/g) was used as the solid support. Standard couplings of amino acids were carried out at 0.125 M in DMF using DIC/OxymaPure^®^ activation (the synthesis method used is optimized by this activator according to Liberty Blue™ recommended operation (CEM Corp., Matthews, NC, USA)), and the corresponding amino acid. Fmoc removal was done with three different reagents: 20% *v*/*v* 4MP in DMF; 20% *v*/*v* PP in DMF, and PZ 10% *w*/*v* in 9:1 DMF/ethanol. It should be noted that due to its solubility PZ is used in lower concentration compared with the other two reagents, and also ethanol should be added to improve its solubility.

Deprotection and coupling were performed following a microwave method described in Table 6. For arginine, an additional coupling step was performed. After completing synthesis in the Liberty Blue™ synthesizer (CEM Corp., Matthews, NC, USA), peptides were cleaved from the resin manually by using TFA under gentle agitation over a period of 2 h at r.t. in the presence of scavengers (standard cleavage solution: TFA/TIS/Water 95:2.5:2.5); for peptides with Trp: TFA/TIS/Water/DOT (92.5:2.5:2.5:2.5) to avoid oxidation. After filtration the crude peptides were precipitated by the addition of cold diethyl ether, centrifuged, washed with cold Et_2_O five times, dried, dissolved in ultrapure water, frozen, and lyophilized.

Three parameters were defined to calculate the yield and purity of the product obtained:

(1) Total crude yield calculated as the percentage of total crude obtained versus the theoretical maximum attainable (0.1 mmol according the scale used in the Liberty Blue synthesizer).
(2) Purity calculated automatically by integrating the area under the curve for the main peak, assigned upon identification by mass spectrometry, in the chromatogram (Tables S1–S4).
(3) Peptide-specific yield calculated as the ratio between the obtained weight in mg with the theoretical one, multiplied by the purity (%area) of the main peak.

### 4.3. Enrichment of the Main Product

Crude peptides were fractionated using preparative Clean-Up^®^ CEC18153 extraction columns (UCT, Bristol, PA, USA) by washing the column twice with methanol and twice with water, loading 10 mg of the peptide dissolved in water onto the column, and eluting successive fractions with 10%, 15%, 20%, 25%, 30%, 40%, and 60% (*v*/*v*) acetonitrile:water. Fractions obtained were evaporated using a Savant SPD 1010 SpeedVac Concentrator (Thermo Fisher Scientific, Asheville, NC, USA). Fractions were evaluated by HPLC, ESI-MS, and MALDI-TOF MS, and CD spectroscopy, for determining the main fraction containing the expected peptide.

### 4.4. Characterization

#### 4.4.1. HPLC

Analyses of synthetic peptides were carried out by Reversed Phase-HPLC (JASCO Corp., Tokyo, Japan) on a XBridge™ BEH C18 column (100 × 4.6 mm, 3.5 µm) (Water Corp., Milford, MA, USA) with a solvent B (acetonitrile with 0.05% TFA) versus solvent A (water with 0.05% TFA) 0%–70% gradient, at a flow rate of 1 mL/min for 8 min. Chromatograms were obtained using ChromPass Chromatography Data System software (Version 1.7.403.1, JASCO Corp., Tokyo, Japan), measured at 214 nm. Additional runs with the peptides using 10%–50% (NBC 155, 759) and 20%–45% (NBC 112, 1951) gradients were performed to improve the resolution in order to collect individual peaks for MS identification.

#### 4.4.2. Mass Spectrometry

Mass spectra of crude synthetic peptides and purified fractions were first obtained in a LCMS-2020 ESI-MS (Shimadzu Corp., Kyoto, Japan), loading 10 µL of 0.1 µg/µL peptide under positive ion mode at 4.5 kV and 350 °C for 15 min. Spectra were recorded and the resulting data analyzed using the Lab Solutions 5.42 SP3 software (Shimadzu Corp.). ESI-MS was performed for monitoring the correct synthesis by identifying various positive *m*/*z* ions corresponding to the product (z = +1, +2, +3, etc.).

Purified/enriched samples were later analyzed by MALDI-TOF MS loading 1 µL of 1 µg/µL peptide and 1 µL of the matrix (10 mg/mL of α-cyano-4-hydroxycinnamic acid in 50% acetonitrile with 0.1% formic acid in water) onto a micro scout sample plate (Bruker Daltonics Inc., Billerica, MA, USA) and measuring in MALDI-TOF-MS Microflex equipment (Bruker Daltonics Inc.) using flexControl 3.0 software (Bruker Daltonik GmbH, Bremen, Germany). Spectra were obtained in positive ion mode by reflection detection as additions of 10 rounds of 30 laser impacts each, at various points of the sample. Individual peaks collected by HPLC with a %area higher than 10% were also analyzed by MALDI-TOF-MS in order to monitor the peptide and its byproducts.

#### 4.4.3. Circular Dichroism Spectroscopy

CD spectroscopy was carried out on a JASCO J-815 CD Spectrometer (JASCO Corp., Tokyo, Japan) in the far ultra-violet (UV) range (190–250 nm), using quartz cuvettes (0.1 cm path length). Each spectrum was recorded averaging three scans in continuous scanning mode. Solvent blank was subtracted from each sample spectrum. Molar ellipticity was calculated for each peptide using 250 µL of 2 mM peptide in 30% (*v*/*v*) 2,2,2-trifluoroethanol. Resulting data were analyzed using Spectra Manager software (Version 2.0, JASCO Corp., Tokyo, Japan).

#### 4.4.4. Theoretical Physicochemical Properties

To complement the experimental results, calculations were done to predict some physicochemical properties. To achieve this, Protparam and Protscale through Expasy server [23] and the peptide calculator through Bachem page [22] were used. Secondary structure was predicted by using Pepfold3 server [20].

### 4.5. Deprotection Kinetics Assay

To assess the deprotection efficiency, we used two amino acid residues, leucine without any side chain protection, and arginine, presenting considerable steric hindrance due to the 2,2,4,6,7-pentamethyldihydrobenzofuran-5-sulfonyl (Pbf) side chain protecting group. Five to ten milligrams of resin with previously-bound Fmoc-l-leucine-OH or Fmoc-l-arginine(Pbf)-OH were weighed and deprotected with 1 mL of each of the aforementioned reagents for 3, 7 and 10 min. The resulting solution was diluted 100-fold with DMF in volumetric flasks and the absorbance of released dibenzofulvene was measured at 300 nm in a JASCO V-630 spectrophotometer (JASCO Corp., Tokyo, Japan). Deprotection efficiency was calculated as the percentage of theoretical resin loading according to Equation (1):

RL= (Fd × Abs)/Ec × l × m
(1)

where Fd = dilution factor, Abs = Absorbance at 300 nm, Ec = extinction coefficient, l = light path length, m = resin weight. The extinction coefficient of DBF at 300 nm was used (7.8 mmol^−1^·cm^−1^) [24].

## 5. Conclusions

In solid phase peptide synthesis the deprotection step is crucial for the quality of the product obtained. The deprotection reagent most commonly used, PP, is sometimes unavailable or its access is difficult (its status is that of a controlled substance regulated by DEA). Therefore, it is convenient to have different options of reagents for that step. This report showed that 4-methylpiperidine and piperazine could be used interchangeably with piperidine; however, there are specific issues to take into account related with the sequence and amino acid residues involved. Beyond the specific results in the deprotection step it is important to consider the specific properties of each reagent, especially in terms of toxicity, where it is possible to establish the order 4MP << PZ < PP (Table S6). Another factor to take into account is the cost-effectiveness for the use of deprotection reagents, for which the following order could be established: PZ > PP > 4MP. Additionally PZ is a solid reagent, which is advantageous for easiness of transportation.

## Figures and Tables

**Table 1:** Synthesized peptide sequences.

Sequence	MW (Da)	Internal ID	Reference
H-FISEAIIHVLHSR-NH_2_	1520.8	NBC112	[14,15]
H-TLEEFSAKL-NH_2_	1036.2	NBC155	[16,17]
H-KKWRWWLKALAKK-NH_2_	1741.2	NBC759	[18]
H-VAPIAKYLATALAKWALKQGFAKLKS-NH_2_	2787.4	NBC1951	[19]

**Table 2:** Yield of crude product, purity, and peptide-specific yield for the synthesized peptides with each deprotection reagent. The highest values are in bold letters. 4MP: 4-methylpiperidine; PP: piperidine; PZ: piperazine.

Peptide	Total Crude Yield (%)			Purity (%)			Peptide-Specific Yield (%)		
	4MP	PP	PZ	4MP	PP	PZ	4MP	PP	PZ
NBC112	**73.2**	72.0	53.6	47.6	43.6	**57.7**	**34.9**	31.3	31.0
NBC155	99.1	98.4	**99.4**	65.1	**83.0**	74.8	64.5	**81.7**	74.3
NBC759	**93.6**	81.6	74.7	50.4	**59.1**	55.6	44.4	**48.2**	41.5
NBC1951	68.5	**79.3**	68.2	20.6	**29.0**	21.4	14.1	**23.8**	14.6

**Table 3:** Summary of the peaks obtained through the ESI-MS analysis for each peptide as presented in the mass spectra of Figure 1.

*m*/*z*	NBC112		NBC155		NBC759		NBC1951	
	Theoretical	Obtained	Theoretical	Obtained	Theoretical	Obtained	Theoretical	Obtained
M+1	1521	1521 *	1036	1036	1741		2787	
M+2	761	761	519	519	872	871	1395	1394
M+3	508	508	346		581	581	930	930
M+4	381		260		436	436	698	698
M+5	305		208		349		559	559

* Observed only with PZ.

**Table 4:** Summary of the MALDI-TOF MS of purified product. HPLC peaks with a %area higher than 10% were analyzed (Figures S1–S4).

Peptide	*m*/*z*	ID	4MP	PP	PZ
NBC112	1520.9	Molecular ion	X	X	X
	1449.8	Alanine deletion	X		X
	1383.7	Histidine deletion	X		X
NBC155	1036.6	Molecular ion	X	X	X
NBC759	1742.1	Molecular ion	X	X	X
	1613.9	Lysine deletion	X	X	X
NBC1951	2787.4	Molecular ion	X	X	X
	2659.3	Glutamine/Lysine deletion	X	X	X

**Table 5:** Representation of sequence hydrophobicity according to Hopp and Woods’ hydrophobicity scale [21], and hydrophilic residues/total residues ratio as calculated via the Bachem web page [22] in square brackets is the ratio divided by the number of residues. Color code for amino acids: basic: blue; acid: red; polar: green; aliphatic: grey; aromatic: purple.

Peptide	#Res	Hopp and Woods Hydrophobicity	Hydrophilic Res/Total Res Ratio
NBC112	13	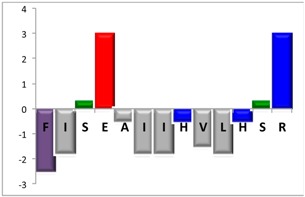	31% [2.38]
NBC155	9	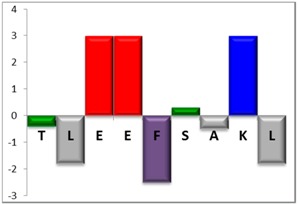	44% [4.89]
NBC759	13	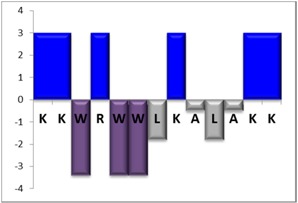	46% [3.54]
NBC1951	26	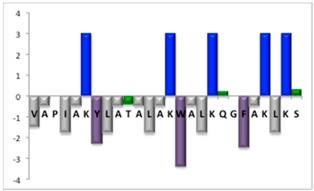	27% [1.04]

**Table 6:** Microwave method for the deprotection and coupling steps.

Step	Temperature (°C)	Power (W)	Hold Time (s)
**Deprotection**	75	155	15
	90	30	50
**Coupling**	75	170	15
	90	30	110

## Supplementary Materials

Supplementary materials can be accessed at: http://www.mdpi.com/1420-3049/21/11/1542/s1.
