# Supplementary material for: Deprotection Reagents in Fmoc Solid Phase Peptide Synthesis: Moving Away from Piperidine?
Source: Molecules. 2016 Nov 15;21(11):1542. doi: 10.3390/molecules21111542 (PMC6274427; doi:10.3390/molecules21111542)
Supplement: Supplementary file 1 [file molecules-21-01542-s001.pdf]

Supplementary Materials: Deprotection Reagents in Fmoc Solid Phase Peptide Synthesis: Moving Away from Piperidine?

Omar F. Luna, Johana Gomez, Constanza Cárdenas, Fernando Albericio, Sergio H. Marshall and Fanny Guzmán

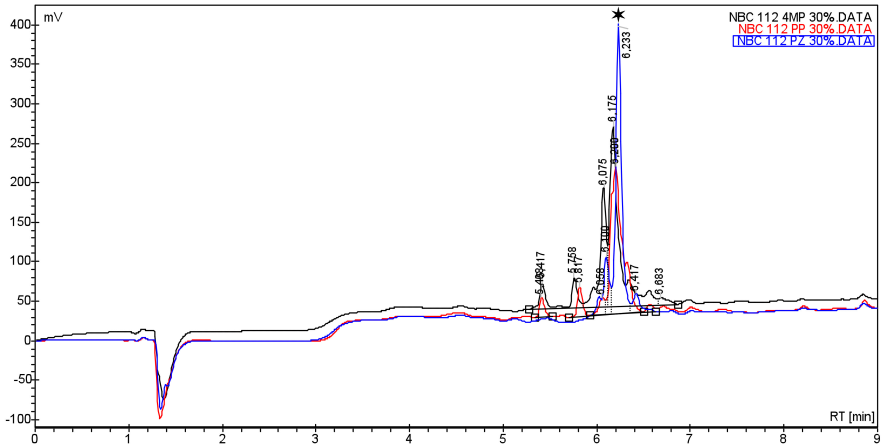

30 % Acetonitrile:Water	4MP		PP		PZ	
**Peak #**	RT [min]	% Area	RT [min]	% Area	RT [min]	% Area
**1**	5.42	5.78	5.41	4.56		
**2**	5.76	5.46	5.82	7.19		
**3**	6.08	26.69	6.06	5.21	6.10	18.18
**4**	6.18	59.67	6.20	83.03	6.23	74.81
**5**	6.68	2.40			6.42	7.01

Figure S1. HPLC for peptide NBC112 30% acetonitrile:water fraction, main specie are indicated with a star. The data are superimposed for the three deprotection reagents; Black: 4-Methyl piperidine (4MP). Red: Piperidine (PP) and Blue: Piperazine (PZ).

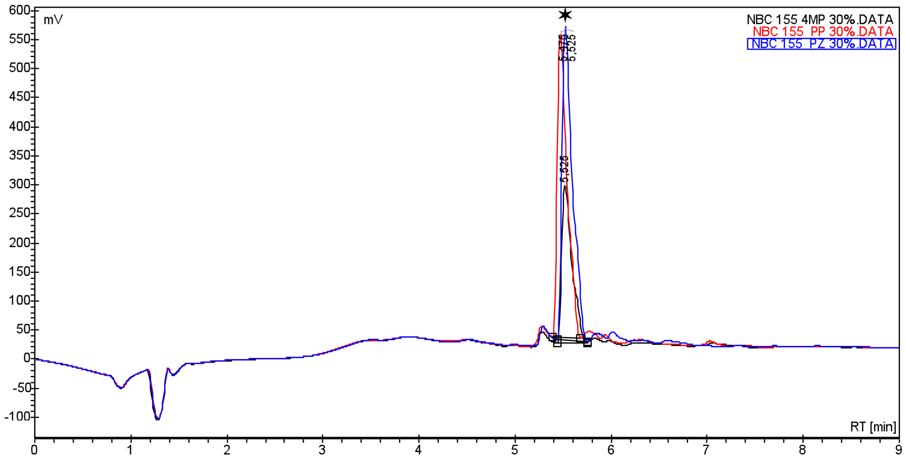

30% Acetonitrile:Water	4MP		PP		PZ	
**Peak #**	RT [min]	% Area	RT [min]	% Area	RT [min]	% Area
**1**	5.53	100	5.48	100	5.53	100

Figure S2. HPLC for peptide NBC155 30% acetonitrile:water fraction, main specie are indicated with a star. The data are superimposed for the three deprotection reagents; Black: 4-Methyl piperidine (4MP). Red: Piperidine (PP) and Blue: Piperazine (PZ).

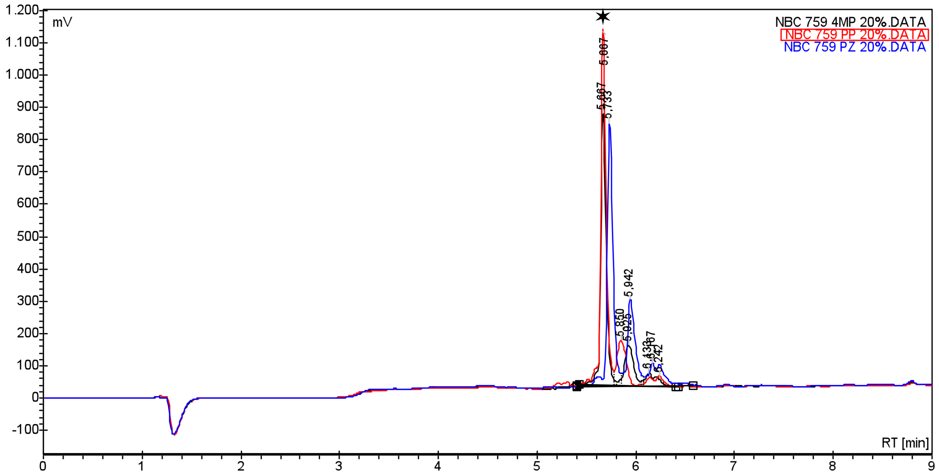

30% Acetonitrile:Water	4MP		PP		PZ	
**Peak #**	RT [min]	% Area	RT [min]	% Area	RT [min]	% Area
**1**	6.59	38.04	6.58	32.30	6.61	34.99
**2**	6.89	50.13	6.89	48.71	6.90	43.42
**3**	7.33	8.89	7.36	10.87	7.30	11.64
**4**	7.74	2.95	7.77	4.99	7.76	9.95
**5**			8.03	3.14		

Figure S3. HPLC for peptide NBC759 20% acetonitrile:water fraction, main specie are indicated with a star. The data are superimposed for the three deprotection reagents; Black: 4-Methyl piperidine (4MP). Red: Piperidine (PP) and Blue: Piperazine (PZ).

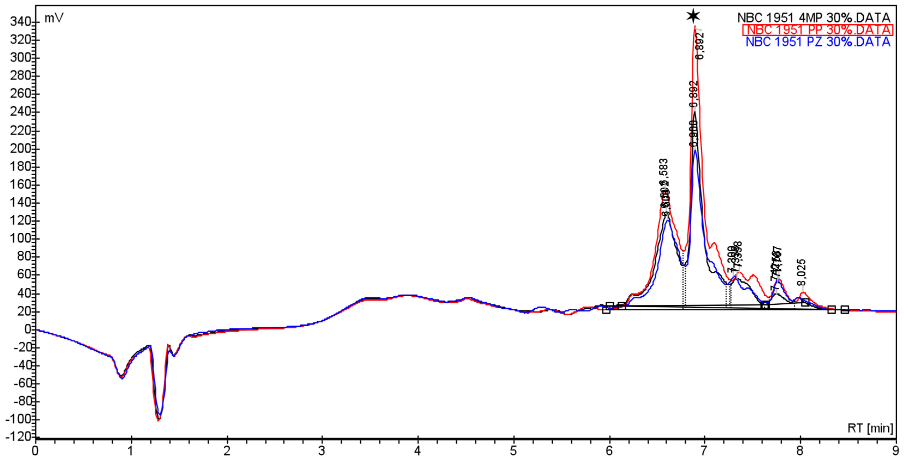

30% Acetonitrile:Water	4MP		PP		PZ	
**Peak #**	RT [min]	% Area	RT [min]	% Area	RT [min]	% Area
**1**	6.59	38.04	6.58	32.30	6.61	34.99
**2**	6.89	50.13	6.89	48.71	6.90	43.42
**3**	7.33	8.89	7.36	10.87	7.30	11.64
**4**	7.74	2.95	7.77	4.99	7.76	9.95
**5**			8.03	3.14		

Figure S4. HPLC for peptide NBC1951 30%acetonitrile:water fraction, main specie are indicated with a star. The data are superimposed for the three deprotection reagents; Black: 4-Methyl piperidine (4MP). Red: Piperidine (PP) and Blue: Piperazine (PZ).

Table S1. Retention times and % area for the crude product with the three deprotection reagents, for NBC112 chromatogram in the figure 1A. The main peak (8) corresponding to the peptide NBC112 is highlighted in grey.

Crude	4MP		PP		PZ	
Peak #	RT (min)	%Area	RT (min)	%Area	RT (min)	%Area
1	4.48	15.71	4.45	9.62		
2					5.08	6.13
3					5.28	4.99
4	5.48	8.60	5.38	11.58	5.38	16.71
5					5.66	1.91
6					5.8	1.01
7	5.93	14.94	5.81	22.41	5.92	5.99
8	6.18	47.65	6.13	43.57	6.18	57.73
9	6.50	13.09	6.44	12.82	6.44	2.72
10					6.56	2.8

Table S2. Retention times and % Area for the crude product with the three deprotection reagents, for NBC155 chromatogram in figure 1B. The main peak (3) corresponding to the peptide NBC155 is highlighted in grey.

Crude	4MP		PP		PZ	
Peak #	RT (min)	%Area	RT (min)	%Area	RT (min)	%Area
1	5.03	6.58	5.00	10.11	4.99	6.72
2	5.34	28.31	5.33	23.85	5.31	26.66
3	5.55	65.11	5.57	66.04	5.53	66.62

Table S3. Retention times and % Area for the crude product with the three deprotection reagents, for NBC759 chromatogram in figure 1C. The main peak (2) corresponding to the peptide NBC759 is highlighted in grey.

Crude	4MP		PP		PZ	
Peak #	RT (min)	%Area	RT (min)	%Area	RT (min)	%Area
1	5.19	5.48				
2	5.66	50.49	5.63	59.09	5.69	55.56
3	5.90	13.90	5.95	13.15	5.93	13.76
4	6.26	14.05	6.26	14.52	6.26	15.63
5	6.53	9.75	6.54	13.24	6.53	15.05
6	6.76	6.33				

Table S4. Retention times and % Area for the crude product with the three deprotection reagents, for NBC1951 chromatogram in figure 1D. The main peak (7) corresponding to the peptide NBC1951 is highlighted in grey.

Crude	4MP		PP		PZ	
Peak #	RT (min)	%Area	RT (min)	%Area	RT (min)	%Area
1	4.85	5.48	4.88	6.59	4.78	6.74
2	5.26	3.65				
3	5.52	10.15	5.62	13.55	5.48	13.48
4	5.92	11.25	5.93	12.45	5.88	11.26
5	6.11	16.52	6.18	14.82	6.13	18.86
6	6.62	24.33	6.67	19.51	6.62	17.58
7	6.93	20.55	6.93	29.04	6.94	21.43
8	7.45	4.44			7.41	6.18
9	7.88	2.33	7.79	2.53	7.79	4.46
10	8.07	1.30	7.99	1.52	6.56	2.8

Table S5. Summary of physicochemical properties calculated. Figures for Pepfold3 predicted structure were made with Chimera [1].

Peptide	#Res	Aliphatic * Index	GRAVY *	IP *	Net Charge pH = 7 **	Average Hydrophilicity **	Pepfold3 Structure
NBC112	13	150	0.777	6.92	1.2	−0.5	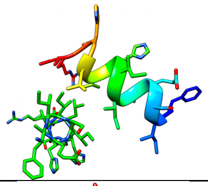
NBC155	9	97.78	−0.022	4.53	0.0	0.3	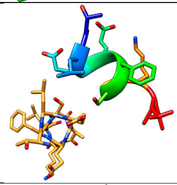
NBC759	13	75.38	−1.192	11.39	7.0	0.2	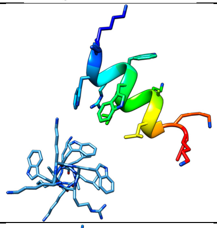
NBC1951	26	113.08	0.408	10.3	6.0	−0.3	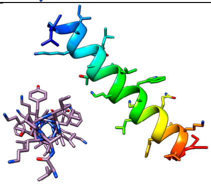

* Calculated through Expasy server; ** Calculatde through Bachem webpage http://www.bachem.com.

Table S6. Summary of physicochemical properties for the deprotection reagents. Extracted from Merckmillipore webpage (http://www.merckmillipore.com/).

Information	4MP	PP	PZ
	**Physicochemical Information**		
CAS Number	626-58-4	110-89-4	110-85-0
Boiling point	105–108 °C (1013 hPa)	106 °C (1013 hPa)	—
Density	0.84 g/cm^3 (20 °C)	0.86 g/cm^3 (20 °C)	1.1 g/cm^3 (20 °C)
Explosion limit	—	1.5%–10.3% (V)	4%–14% (V)
Flash point	13 °C	16 °C	65 °C
Ignition temperature	—	320 °C	320 °C DIN 51794
Melting point	4–5 °C	−10.8 °C	107–111 °C
pH value	13 (100 g/L H_2O 20 °C)	12.6 (100 g/L H_2O 20 °C)	12 (150 g/L H_2O 20 °C)
Vapor pressure	16 hPa (20 °C)	34 hPa (20 °C)	<10 hghjPa (20 °C)
	**Toxicological Information**		
LD 50 dermal	—	LD50 Rabbit 276 mg/kg	LD50 Rabbit 8300 mg/kg
LD 50 oral	—	—	LD50 Rat 2600 mg/kg

References

1. Pettersen, E.F.; Goddard, T.D.; Huang, C.C.; Couch, G.S.; Greenblatt, D.M.; Meng, E.C.; Ferrin, T.E. UCSF Chimera–a visualization system for exploratory research and analysis. *J. Comput. Chem.* **2004**, *25*, 1605–1612.
